# Anovaginal Colonization by Group B *Streptococcus* and *Streptococcus anginosus* among Pregnant Women in Brazil and Its Association with Clinical Features

**DOI:** 10.3390/antibiotics13010085

**Published:** 2024-01-16

**Authors:** Natalia Silva Costa, Laura Maria Andrade Oliveira, Andre Rio-Tinto, Isabella Bittencourt Ferreira Pinto, Ana Elisa Almeida Santos Oliveira, Julia de Deus Santana, Laiane Ferreira Santos, Rayssa Santos Nogueira Costa, Penelope Saldanha Marinho, Sergio Eduardo Longo Fracalanzza, Lucia Martins Teixeira, Tatiana Castro Abreu Pinto

**Affiliations:** 1 Instituto de Microbiologia Paulo de Góes, Universidade Federal do Rio de Janeiro, Rio de Janeiro 21941-902, Brazil; natisilvacosta@gmail.com (N.S.C.); lauraoliveira@micro.ufrj.br (L.M.A.O.); andrertmf@micro.ufrj.br (A.R.-T.); isabittencourtk1@gmail.com (I.B.F.P.); souelisaso@gmail.com (A.E.A.S.O.); juliadedeus550@gmail.com (J.d.D.S.); laianefs@gmail.com (L.F.S.); ray1dsunshinne@gmail.com (R.S.N.C.); fracalanzza@micro.ufrj.br (S.E.L.F.); lmt2@micro.ufrj.br (L.M.T.); 2 Faculdade de Medicina, Maternidade Escola, Universidade Federal do Rio de Janeiro, Rio de Janeiro 22240-000, Brazil; penelope@me.ufrj.br

**Keywords:** group B *Streptococcus*, *Streptococcus agalactiae*, *Streptococcus anginosus*, pregnant women, anovaginal colonization, chromogenic media

## Abstract

*Streptococcus agalactiae* (Group B *Streptococcus*; GBS) is a leading cause of neonatal invasive disease worldwide. GBS can colonize the human gastrointestinal and genitourinary tracts, and the anovaginal colonization of pregnant women is the main source for neonatal infection. *Streptococcus anginosus*, in turn, can colonize the human upper respiratory, gastrointestinal, and genitourinary tracts but has rarely been observed causing disease. However, in the last years, *S. anginosus* has been increasingly associated with human infections, mainly in the bloodstream and gastrointestinal and genitourinary tracts. Although anovaginal screening for GBS is common during pregnancy, data regarding the anovaginal colonization of pregnant women by *S. anginosus* are still scarce. Here, we show that during the assessment of anovaginal GBS colonization rates among pregnant women living in Rio de Janeiro, Brazil, *S. anginosus* was also commonly detected, and *S. anginosus* isolates presented a similar colony morphology and color pattern to GBS in chromogenic media. GBS was detected in 48 (12%) while *S. anginosus* was detected in 17 (4.3%) of the 399 anovaginal samples analyzed. The use of antibiotics during pregnancy and history of urinary tract infections and sexually transmitted infections were associated with the presence of *S. anginosus*. In turn, previous preterm birth was associated with the presence of GBS (*p* < 0.05). The correlation of GBS and *S. anginosus* with relevant clinical features of pregnant women in Rio de Janeiro, Brazil, highlights the need for the further investigation of these important bacteria in relation to this special population.

## 1. Introduction

*Streptococcus agalactiae*, also referred to as Group B *Streptococcus* (GBS), is a leading cause of neonatal morbidity and mortality worldwide, with estimates of 392,000 neonatal disease cases, 91,000 neonatal deaths, 46,000 stillbirths, and 518,000 preterm births each year [1]. GBS is frequently found colonizing the human gastrointestinal and genitourinary tracts, with 10–30% of pregnant women being colonized by this microorganism. GBS anovaginal colonization is a risk factor for neonatal invasive disease, such as sepsis and meningitis [2,3]. GBS can be vertically transmitted to the fetus before delivery through in utero acquisition or to the newborn during labor [4].

The *Streptococcus anginosus* group (SAG), including *Streptococcus anginosus*, *Streptococcus constellatus*, and *Streptococcus intermedius*, is commonly found in the human oral cavity but also in other regions such as the oropharyngeal, gastrointestinal, and genitourinary tracts. The SAG has been considered part of the commensal microbiota, eventually associated with dental abscesses and periodontal diseases, but their pathogenic potential has been vastly underestimated [5]. However, the SAG can cause life-threatening infections, with incidence rates higher than those for *Streptococcus pyogenes* and *Streptococcus agalactiae* combined [6,7,8,9]. The number of cases of severe infections caused by SAG bacteria, in both adults and children, has risen over the years, and the SAG is now considered an emerging pathogen instead of a simple commensal species of the human microbiota.

The species *S. anginosus* can express group A, C, F, and G Lancefield antigens or none at all [10,11]. It is mainly isolated from blood cultures and gastrointestinal and genitourinary tract infections and is often a component of polymicrobial infections in patients with oral, head, neck, and abdominal abscesses [6,12,13]. In addition, *S. anginosus* is one of most common pathobionts identified in patients with aerobic vaginitis [14]. Although the influence of *S. anginosus* in aerobic vaginitis and its role in the vaginal tract during pregnancy are still unknown, there have been reports of fatal neonatal sepsis due to this microorganism [15,16].

Although both GBS and *S. anginosus* can colonize the gastrointestinal and genitourinary tracts of pregnant women and be associated with disease in the perinatal period, anovaginal screening during pregnancy is usually only aimed at GBS, and data regarding the prevalence of *S. anginosus* in this population are still scarce. During an active surveillance aimed at GBS anovaginal screening among pregnant women living in Rio de Janeiro, Brazil, we noticed that *S. anginosus* anovaginal colonization was also common in the population analyzed and that *S. anginosus* isolates can show a colony morphology and color pattern similar to those of GBS in chromogenic media. Thus, here, we assessed the prevalence of anovaginal colonization by GBS and *S. anginosus* among a cohort of pregnant women living in Rio de Janeiro, Brazil, and evaluated the correlation of the microbiological results with clinical and sociodemographic data.

## 2. Results

**Figure 1:**
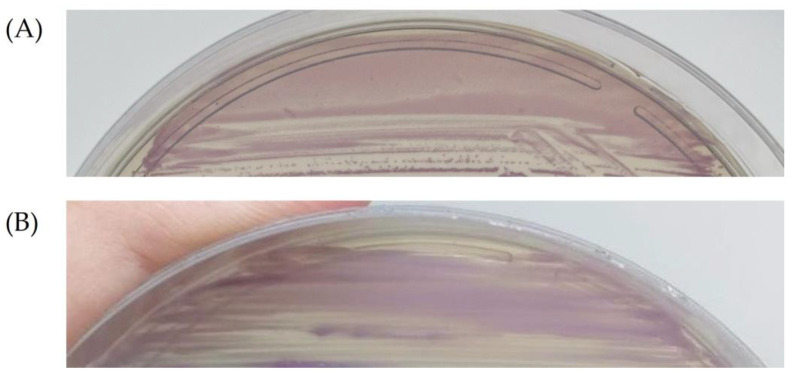
(**A**) Group B *Streptococcus* strain growing in CHROMagar™ StrepB medium showing colonies with mauve color. (**B**) *S. anginosus* strain growing in CHROMagar™ StrepB medium showing colonies with mauve coloration and similar appearance to GBS.

All 399 anovaginal samples were simultaneously processed using both sheep blood agar and CHROMagar™ StrepB. Group B *Streptococcus* (GBS) was detected in 48 (12%) of the 399 anovaginal samples analyzed. Among these, 13 (27.1%) were detected in both media (sheep blood agar and CHROMagar™ StrepB), while 23 (47.9%) were detected only in CHROMagar™ StrepB, and 12 (25%) were detected only in sheep blood agar. All 48 GBS strains were beta-hemolytic and reacted with Lancefield’s group B antiserum. All 36 GBS strains recovered from CHROMagar™ StrepB showed the expected mauve appearance (Figure 1A).

Besides the GBS-positive anovaginal samples, another 75 samples yielded colonies that showed a coloration similar to that of GBS in CHROMagar™ StrepB but were not identified as GBS via MALDI-TOF MS. A total of 15 bacterial species of Gram-positive cocci were identified among these 75 clinical samples, including *Enterococcus faecalis* (12), *Streptococcus salivarius* (10), *Streptococcus oralis* (9), *Enterococcus faecium* (9), *Lactococcus garviae* (8), *Staphylococcus epidermidis* (7), *Aerococcus viridans* (6), *Streptococcus parasanguinis* (5), *Streptococcus mitis* (5), *Staphylococcus hominis* (3), *Weisella confusa* (2), *Streptococcus infantis* (1), *Enterococcus durans* (1), *Staphylococcus condimenti* (1), and *Weisella cibaria* (1). However, one streptococcal species stood out among the non-GBS mauve colonies, namely, *S. anginosus*.

*S. anginosus* was detected in 17 (4.3%) of the 399 anovaginal samples analyzed. All 17 *S. anginosus* strains were identified using MALDI-TOF MS with scores ≥2.0, indicating high reliability for species identification. The majority of *S. anginosus* strains (*n* = 13, 76.5%) were detected only in CHROMagar™ StrepB plates, and they all showed mauve coloration and a similar appearance to GBS (Figure 1B). The remaining four strains (23.5%) were detected only in the sheep blood agar plates. An anovaginal sample from one patient contained both GBS and *S. anginosus*; in this case, GBS was recovered from sheep blood agar, and *S. anginosus* was recovered from CHROMagar™ StrepB.

Only three (17.6%) *S. anginosus* strains were beta-hemolytic. Most strains (*n* = 14, 82.4%) did not react with any of the Lancefield’s group antisera tested, while two belonged to group G and one belonged to group F. The group G *S. anginosus* isolates were beta-hemolytic, while the group F *S. anginosus* strain was non-hemolytic. The *S. anginosus* strain co-isolated with GBS was non-hemolytic and did not react with any Lancefield’s group antisera.

Regarding the clinical and sociodemographic aspects evaluated, the presence of GBS and *S. anginosus* was associated with arterial hypertension (Table 1). Previous cases of urinary tract infection (UTI) and sexually transmitted infections (STIs) and the use of antibiotics during pregnancy were more common in *S. anginosus*-positive women and less common in GBS-positive women when compared to those negative for both. In contrast, previous preterm birth was more common in GBS-positive women and less common in *S. anginosus*-positive women (Table 1).

## 3. Discussion

GBS is a main agent involved in invasive infections in newborns, accounting for a high burden of young infant mortality, in addition to causing diseases in pregnant women and postpartum women and stillbirths worldwide [2]. GBS neonatal infections are classified as prenatal-onset, early-onset, or late-onset diseases according to the transmission mode (vertical or horizontal) and the timing of the onset of symptoms (before delivery, up to 7 days after birth, and between 7 and 90 days after birth) [4]. GBS colonization rates usually vary according to geographical area. Reports of distinct Brazilian regions show rates of GBS colonization among pregnant women ranging from 10% to 32% [17,18,19]. In fact, the GBS colonization rate reported in this study agrees with the 10.8% rate recently reported in Rio de Janeiro, Brazil [20].

While GBS is a recognized agent of invasive neonatal infections worldwide, *S. anginosus* is an emerging human pathogen. *S. anginosus* has been associated with pyogenic and systemic infections at any anatomical site in the body, including intra-abdominal areas, the urogenital area, the liver, lungs, the heart, the brain, skin, and soft tissues, with the formation of empyemas and abscesses [6,7,8,21]. *S. anginosus* is mainly isolated from blood cultures and gastrointestinal and genitourinary tract infections and is often present as part of a polymicrobial infection in patients with oral, head, neck, and abdominal abscesses [6,13,21].

Although there are no robust data on the prevalence of *S. anginosus* in anovaginal specimens from pregnant women, in other studies, *S. anginosus* was one of the most prevalent specimens in vaginal or female urogenital microbiota, including during pregnancy and postpartum, associated with urinary symptoms [22,23,24]. Likewise, *S. anginosus* has been associated with urgency urinary incontinence [25,26]. A few studies have reported *S. anginosus* in more than 70% of genitourinary tract infections among 3–10 year-old children [27,28].

*S. anginosus* has been the source of much controversy and confusion regarding taxonomy and classification. This species can be alpha, beta-, or nonhemolytic. Likewise, *S. anginosus* may or may not have A, C, F, or G Lancefield group antigens. According to the US Centers for Disease Control and Prevention (CDC), most *S. anginosus* strains have the group F antigen or no antigen of this type and only rarely present group A, C, or G antigens [29]. Indeed, most (82.4%) of the *S. anginosus* strains analyzed in this study did not present any of the Lancefield antigens, and only three strains presented G and F group antigens (two strains and one strain, respectively). However, more recent technologies, such as MALDI-TOF MS, have been aiding the fast and accurate identification of such species [30]. Here, all *S. anginosus* strains were identified using MALDI-TOF MS, with scores indicating high reliability, suggesting that this technique might be a good option for *S. anginosus* identification.

Among the 48 GBS recovered from anovaginal samples, 13 (27.1%) were detected in both media (sheep blood agar and CHROMagar™ StrepB), while among the 17 *S. anginosus* samples recovered, 13 (76.5%) were detected in CHROMagar™ StrepB and 4 were found in sheep blood agar. The fact that not all the strains were identified in both culture media does not mean that those strains are not capable of growing on them. In fact, it is possible that a strain present in a clinical sample may be stressed or have low cell numbers in comparison to other accompanying bacteria, impairing the recovery of said strain from a specific medium during the primary isolation.

In this study, use of antibiotics during pregnancy and a previous UTI and/or STI were more common in *S. anginosus*-positive women and less common in GBS-positive women when compared to the negative ones. In previous studies, *S. anginosus* was the predominant microorganism in patients with aerobic vaginitis [14,31,32]. It was hypothesized that *S. anginosus* causes purulent discharge and vaginal wall reddening in patients with aerobic vaginitis, which are associated with damage to the vaginal epithelial wall. This previously established association of *S. anginosus* with bacterial vaginitis and the resulting tissue damage may help to explain, at least in part, why *S. anginosus* was positively correlated with STIs in this study since it is known that both vaginitis status and tissue damage can contribute to STI transmission. Moreover, *S. anginosus* can form biofilms in bacterial vaginitis communities [33], making it difficult to restore a healthy environment and leading to recurrent bacterial vaginitis, thereby increasing the chances of contracting an STI [34,35]. In turn, use of antibiotics has been associated with modifications of the microbiota and thus with an increased risk of developing bacterial vaginitis. In general, GBS seems to be more susceptible to antibiotics than *S. anginosus* strains, which would render the use of antibiotics a selective pressure towards *S. anginosus*.

On the other hand, only GBS was associated with previous preterm birth in the present study. Similarly, other studies conducted in Brazil have reported an association between GBS and clinical aspects, such as UTIs and vaginal discharge [19,36]. Indeed, GBS has been known to be the cause of many prenatally related outcomes, including not only preterm birth but also stillbirth and miscarriage [2].

*S. anginosus* is rarely considered a pathogen as it constitutes the commensal microbiota of the upper respiratory, digestive, and reproductive tracts. However, the increasing reports of opportunistic infections caused by this species indicates that *S. anginosus* should be considered in the clinical diagnosis and treatment of related infections, highlighting its potential relevance for maternal and infant health. Despite GBS being a recognized neonatal pathogen, this species is frequently overlooked during antenatal care, especially in countries without a national policy for GBS screening, like Brazil. Thus, our results indicate the need for the continuous surveillance of GBS and *S. anginosus* among pregnant women in the analyzed setting as well as the further investigation of the clinical association between *S. anginosus* and adverse pregnancy outcomes.

## 4. Materials and Methods

### 4.1. Population Included in the Study

A total of 399 pregnant women between 35 and 37 gestational weeks, attended to at the Teaching Maternity of Universidade Federal do Rio de Janeiro (UFRJ) between September 2021 and September 2022, were enrolled in the study. The project was approved by the local research ethics committee under number 43389321.9.0000.5257, and written informed consent was obtained from all participants. In addition, clinical and sociodemographic information were collected through a questionnaire, which included age, marital status, region of birth, ethnicity, education level, pre-existing pathologies, history of sexually transmitted infections (STI), presence of vaginal discharge, previous cases of urinary tract infection (UTI), use of antibiotics, vaccines administered during pregnancy, previous preterm delivery, previous miscarriage, previous neonatal infection, and previous stillbirth.

### 4.2. Collection of Clinical Specimens and Detection of GBS and S. anginosus

Anovaginal specimens were collected as previously described [20], maintained in Amies transport media, and immediately forwarded to our laboratory. Aliquots of the Amies medium containing the swabs were subjected to a pre-enrichment step in Todd-Hewitt broth (Sigma-Aldrich, St. Louis, MI, USA) supplemented with nalidixic acid (15 μg/mL; Sigma-Aldrich) and gentamicin (8 μg/mL; Sigma-Aldrich). The pre-enrichment cultures were incubated for 24 h at 37 °C; then, the cultures were streaked onto sheep blood agar plates (PlastLabor, Rio de Janeiro, Brazil) and incubated for 24 h at 37 °C under 5% CO2 atmosphere. Simultaneously, another aliquot of Amies was directly streaked onto CHROMagar™ StrepB (CHROMagar, Paris, France). The identification of hemolytic and mauve-colored colonies, for the colonies recovered from sheep blood agar and chromogenic media, respectively, was performed using MALDI-TOF MS (Bruker Microflex LT, Bruker Daltonics, Bremen, Germany) in accordance with the manufacturer’s instructions. Hemolytic colonies identified as *Streptococcus* sp. via MALDI-TOF MS were also subjected to determination of Lancefield groups using a commercial latex agglutination test (Pastorex™ Strep, Bio-Rad Laboratories, Hercules, CA, USA) in accordance with the manufacturer’s recommendations.

### 4.3. Statistical Analysis

Data were analyzed using Fisher’s exact test or Chi-square test using GraphPad Prism software version 9.3.1 (GraphPad Software, Boston, MA, USA). *p*-values of <0.05 were considered statistically significant.

## 5. Conclusions

The present study showed that *S. anginosus* was the second-most-common species found among anovaginal specimens of pregnant women living in Rio de Janeiro, Brazil, presenting characteristics similar to GBS in chromogenic media. The presence of *S. anginosus* was associated with history of urinary tract infections and sexually transmitted infections as well as the use of antibiotics during pregnancy, while previous preterm birth was associated with the presence of GBS. The presented data highlight the need for additional studies considering these relevant bacteria among pregnant women in Rio de Janeiro, Brazil.

## Figures and Tables

**Table 1:** Distribution of clinical and sociodemographic aspects according to the presence or absence of *S. agalactiae* (GBS) and *S. anginosus* (SAG) in anovaginal specimens recovered from the 399 pregnant women analyzed. Total number of responses for each question varies.

Clinical or Sociodemographic Aspect	Absence of GBS and SAG (327) %Positive (*n*)	Presence of GBS (47) %Positive (*n*)	Presence of SAG (17) %Positive (*n*)	*p*-Value
Mean age	30.4	31.2	32.4	N/A
Median age	30	31	32	N/A
Age standard deviation	6.9	6.3	7.1	N/A
Black/brown ethnicity	61.8% (201/305)	72.3% (34/48)	68.7% (11/16)	0.3001
White ethnicity	33.4% (102/305)	25% (12/48)	31.2% (5/16)	0.5321
Diabetes	48.7% (152/312)	53.2% (25/48)	64.7% (11/17)	0.0602
Arterial hypertension	20.2% (63/312)	31.9% (15/48)	41.2% (7/17)	**0.0056**
Hypothyroidism	5.1% (16/312)	8.5% (4/48)	11.8% (2/17)	0.1990
Sexually transmitted infection	9.8% (30/306)	6.4% (3/48)	18.7% (3/16)	**0.0135**
Vaginal discharge	23.9% (74/310)	29.8% (14/48)	29.4% (5/17)	0.5967
Urinary tract infection	30.4% (95/312)	17.0% (8/48)	56.2% (9/16)	**<0.0001**
Use of antibiotics	38.2% (118/309)	27.6% (13/48)	50.0% (8/16)	**0.0060**
Previous preterm birth	10.7% (33/309)	19.1% (9/48)	5.9% (1/17)	**0.0170**
Previous miscarriage	22.9% (71/309)	31.9% (15/48)	23.5% (4/17)	0.2458
Previous stillbirth	8.7% (27/309)	4.2% (2/48)	5.9% (1/17)	0.3438

N/A—not applicable, bold—statistically significant *p*-Values

## Data Availability

The data presented in this study are available in the present article. Any additional information can be made available on request made to the corresponding author.
